# Diagnostics in Pleural Disease

**DOI:** 10.3390/diagnostics10121046

**Published:** 2020-12-04

**Authors:** Anand Sundaralingam, Eihab O. Bedawi, Najib M. Rahman

**Affiliations:** 1 Oxford Centre for Respiratory Medicine, Oxford University Hospitals NHS Foundation Trust, Oxford OX3 7LE, UK; Eihab.Bedawi@ouh.nhs.uk (E.O.B.); najib.rahman@ndm.ox.ac.uk (N.M.R.); 2 Oxford Respiratory Trials Unit, University of Oxford, Oxford OX3 7LE, UK; 3 Oxford NIHR Biomedical Research Centre, Oxford OX4 2PG, UK

**Keywords:** pleural effusion, pleural fluid analysis, image-guided pleural biopsies, thoracoscopy, thoracic ultrasound, malignant pleural mesothelioma

## Abstract

Pleural disease diagnostics represent a sprawling topic that has enjoyed a renaissance in recent years from humble beginnings. Whilst pleural patients are heterogeneous as a population and in the aetiology of the disease with which they present, we provide an overview of the typical diagnostic approach. Pleural fluid analysis is the cornerstone of the diagnostic pathway; however, it has many shortcomings. Strong cases have been made for more invasive upfront investigations, including image-guided biopsies or local anaesthetic thoracoscopy, in selected populations. Imaging can guide the diagnostic process as well as act as a vehicle to facilitate therapies, and this is never truer than with the recent advances in thoracic ultrasound.

## 1. Introduction

Patients with pleural disease represent a heterogenous population and whilst there is not a “one size fits all” approach to diagnosis or management, we provide an overview of the typical diagnostic approach and recent developments that have furthered our understanding of diagnostics within pleural disease. The vast majority of patients with pleural disease will present with a pleural effusion. Therefore, the main focus of this review will pertain to diagnostics within this context; however, an approach to investigating pleural thickening will also be explored. Patients with pleural effusion will often present with symptoms of dyspnoea, chest pain, or cough but with the increasing utilisation of cross-sectional imaging, effusions and thickening are often identified incidentally, in asymptomatic patients.

Estimates suggest that 0.26 mL of fluid per kg of body weight is contained within each pleural cavity (approximately 18 mL in a 70 kg adult) [1,2,3]. Pleural fluid (PF) is produced by the parietal pleura and reabsorbed via parietal pleural lymphatic channels. This homeostasis is dependent on the balance of hydrostatic and oncotic pressures between the systemic and pulmonary circulation and the pleural space itself [4]. Disruptions to this homeostasis often underpin “transudative” effusions, whilst it is traditionally thought that “exudative” effusions result from an increase in the permeability of the pleural membranes and microvasculature [5]. In health, the parietal pleural lymphatic channels are capable of increasing their flow rate and re-absorption by a factor of 20 [4]. It is therefore postulated that in addition to allowing for frictionless sliding of the pleural membranes during the respiratory cycle, the pleural space serves as an extrapulmonary reservoir for pulmonary oedema arising from the pulmonary interstitium, in order to minimise interference with gas exchange [6,7]. Pleural effusion can therefore be thought of as a state of excessive production that overwhelms the usual mechanisms for resorption, a disruption to the usual mechanisms of resorption, or a combination of the two [8].

With over 60 documented causes for pleural effusions identified in the literature, with some examples included in Table 1, no single test is likely to ever provide the entire diagnosis [9]. A combination of history, physical examination, laboratory tests, and radiology is essential in securing a diagnosis.

## 2. Pleural Fluid

PF analysis remains the cornerstone in diagnosing pleural effusions of unknown aetiology. It is however important to note that if the clinician is able to satisfactorily achieve their diagnosis based on history, examination findings, and radiology and hence, PF analysis would not affect management of the patient, thoracocentesis as a first line investigation is not required. This is never more applicable than in the context of congestive cardiac failure. Pleural effusion associated with heart failure has an estimated annual incidence in the USA of 500,000 cases and remains one of the commonest causes of pleural effusion worldwide [11]. Whilst traditionally thought to only present with bilateral effusions, this was the case in only 58% across a large case series covering 3245 consecutive patients, with 27% appearing on the right only and 14% on the left [12].

The British Thoracic Society (BTS) advocates against pleural aspiration, where there is a strong pre-test probability for a transudative cause with typical features (e.g., bilateral effusions, responding to therapies) [13]. However, it is increasingly recognised that a significant proportion of patients presenting with a pleural effusion will have dual pathology driving their presentation and this bears consideration. In a prospective study of 126 patients with pleural effusion of unknown aetiology, 30% (38/126) were found to have more than one cause for their effusion, of which the commonest secondary cause was congestive cardiac failure [5,14].

Where the diagnosis remains in doubt or when a transudative cause of pleural effusion is behaving atypically, PF analysis forms the next step in assessment. Table 2 highlights the tests that all PF specimens should be analysed for, generally considered the minimum standard following initial thoracocentesis.

## 3. Pleural Fluid Biochemistry

It is likely the PF biochemistry panel will be the first to return and thereby, initially guide the diagnostic pathway. Based on the PF biochemistry, the first decision to make is whether the PF represents a transudate or exudate. Dr Light’s 1972 criteria for differentiating transudative from exudative effusions, based on the PF biochemistry across 150 effusions he sampled prospectively, retain clinical utility even today. These criteria classified an exudate as meeting one or more criteria of: a PF protein to serum ratio of greater than 0.5, a PF lactate dehydrogenase (LDH) to serum ratio of greater than 0.6, or a PF LDH of greater than 200 IU [15]. This latter criterion was later modified to greater than two-thirds of the upper limit of the normal LDH level [16]. These criteria have been shown to predict an exudate correctly with a diagnostic accuracy rate of 94.7% in some series [17].

It should be noted, however, that these criteria are skewed towards “overcalling” an exudate—the so called “pseudo-exudate”. This is such that some 27% of cases of heart failure may be classified as an exudate, an effect that is exaggerated when prior diuretic therapy has been commenced [18]. In this situation, the serum to PF albumin difference (>12 g/L) or serum to PF total protein difference (>31 g/L) can be used to correctly reclassify the effusion as a transudate [19]. One benefit of “overcalling” is that it is less likely to miss important causes for exudative effusions, such as malignancy. Nonetheless, case series have demonstrated that between 1 and 10% of malignant pleural effusions (MPE) are characterised as transudates, despite using Light’s criteria [20,21].

It is worth mentioning the issue of “discordant PF biochemistry”, for example when the protein criteria suggest an exudate whilst the LDH criteria a transudate and vice versa. In one case series, up to 29% (229/792) of pleural effusions classified as an exudate were discordant. The discordant pleural effusions were seen in an older population (75 years vs. 70 years) and where the diagnosis included global fluid overloaded states (11% vs. <2%) [22]. This once again highlights the role of dual pathologies contributing to pleural effusion in selected populations and the difficulties this poses in the classification of pleural effusions by conventional methods [8]. Another area that poses a diagnostic challenge is in differentiating an inflammatory malignant effusion from pleural infection. By definition, this is a patient group that does not lend itself easily to study within the setting of a randomised controlled trial. Procalcitonin has been proposed as a specific marker of bacterial infection and has been used as a differentiator from other states of systemic inflammation in other infections [23]. However, procalcitonin showed no statistically significant difference in diagnostic utility when compared to C-reactive protein (CRP) or white cell count, in a study by Dixon et al. across 425 patients of whom 80 had pleural infection [24]. Whilst this finding was somewhat unexpected, given prior smaller studies, it may yet have a role in a specific subset of patients hitherto undefined.

PF glucose and pH will often be immediately available, as a point of care test, and may determine the need for immediate management in some conditions (e.g., pleural infection). This analysis is subject to error if the sample is exposed to air or local anaesthetic and so care must be taken in processing. A PF pH < 7.2 and glucose < 3.4 mmol/L have been shown to be reliable in differentiating between a complicated parapneumonic pleural effusion (CPPE) which requires immediate tube thoracostomy drainage and an uncomplicated parapneumonic pleural effusion (UPPE) [25,26,27]. A recent large (*n* = 2971), retrospective database study concluded that whilst the relationship between PF pH and glucose was non-linear, there was concordance between the two in producing clinically meaningful outcomes. They conclude that either test on its own will provide the necessary information in 90% of cases, but they recommend exercising caution in patients with a baseline hyperglycaemic state, which may have resulted in discordance between the two (i.e., low PF pH, normal/high PF glucose) [28]. It should be borne in mind that whilst these criteria are highly sensitive at identifying CPPE, it is not specific to pleural infection and there are a number of other causes of pleural effusion that may yield similar results. Of note, it has been shown that PF pH can vary even within the same patient, depending on which locule is sampled within a multiloculated effusion [29]. As with any test, PF biochemistry must be interpreted in the appropriate clinical context. Table 3 highlights some typical PF biochemical features with their corresponding clinical conditions.

Novel PF biomarkers are always on the horizon; however, few have made it into routine clinical practice. PF adenosine deaminase (ADA), a purine-degrading enzyme found in T-lymphocytes, has been shown to have a strong negative predictive value in excluding pleural TB in low-incidence areas (NPV 99% when < 30 IU/L) [30]. However, false positives in empyema, rheumatoid pleuritis, and malignancy are seen and therefore, reserving its use for only lymphocytic effusions may increase its positive predictive value [13]. In higher incidence areas, an LDH:ADA ratio may be more specific and the combination of ADA and unstimulated interferon-gamma (IFN-γ) in PF, which has been shown to be superior to ADA in all parameters, may be more helpful if accessible [31,32].

In the context of pleural infection, PF soluble urokinase Plasminogen Activator Receptor (suPAR) has been shown to be potentially of use in predicting the need for tube thoracostomy in parapneumonic effusions, though prospective validation in a multicentre trial setting is awaited [33].

## 4. Pleural Fluid Microscopy Culture and Sensitivity (MCS)

**Figure 1:**
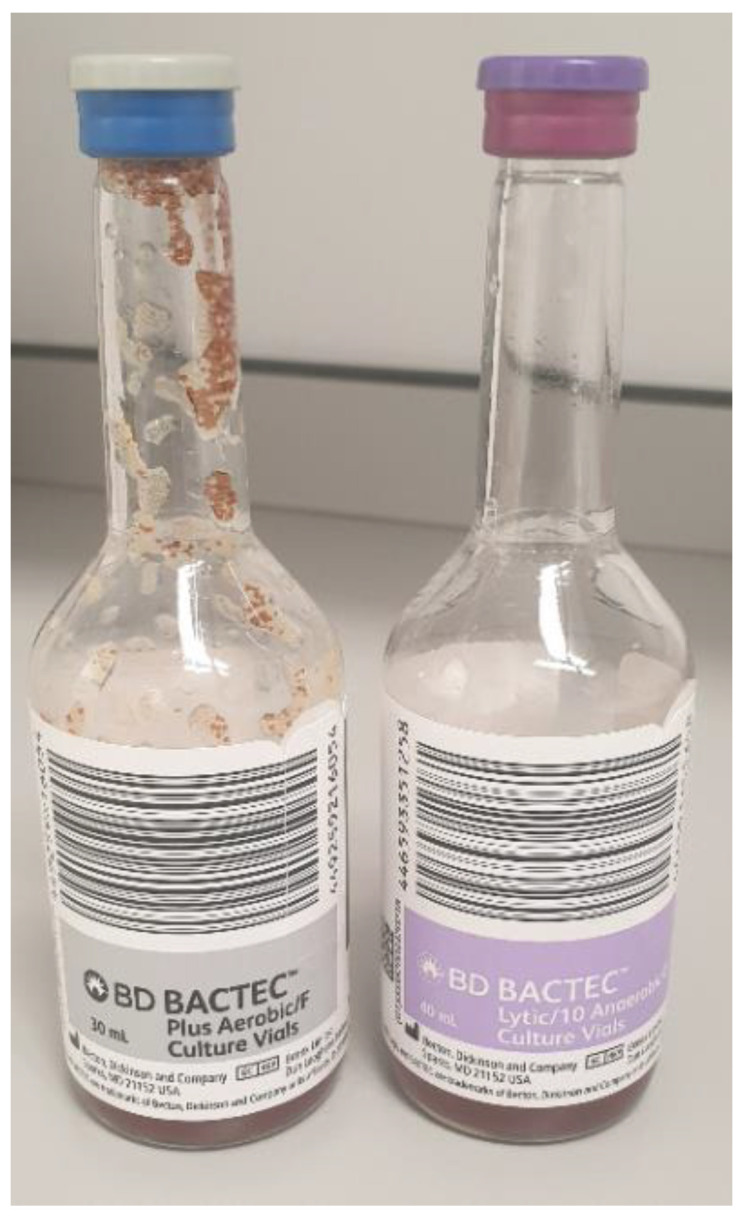
BACTEC bottles.

Whilst the PF biochemistry may be suggestive of pleural infection, the gold standard for diagnosing the condition is with positive microbiological growth within the PF. Unfortunately, yields from PF are quite poor; a recent systematic review across 75 studies suggests that PF culture is only positive in 56% of cases [36]. This yield may be improved further by inoculating the PF into enrichment medium [37]. The yield from PF culture for *Mycobacterium tuberculosis* has traditionally been even worse (quoted as 10–20%) [13,38,39]. However, the use of liquid culture medium (BACTEC, Figure 1) and inoculation by the bedside has been observed to have greater yields (63% sensitivity) with a reduction in time to positive culture [40,41].

A number of factors influence microbiological yield from PF, including the prior usage of antibiotics, polymicrobial infection not accurately represented through standard microscopy and culture techniques, and infection with fastidious organisms not easily grown in culture medium. With the use of next generation techniques including 16S ribosomal RNA (rRNA) sequencing and whole genome sequencing (WGS) alongside accelerated diagnostic pathways (see core cutting needle biopsies), some of these deficits may be overcome.

## 5. Pleural Fluid Cytology

Cell differentials in PF are a useful adjunct in diagnosing the underlying aetiology, though it must be noted that it is neither specific nor sensitive. Lymphocyte predominant effusions (>50%) are most often associated with malignancy, congestive cardiac failure, and tuberculosis, of which the latter sees particularly high levels [8,13]. Generally, any chronic effusion will eventually produce a lymphocytic effusion. Neutrophilic effusions are seen in more acute disease processes such as in a parapneumonic effusion or pulmonary embolism, though 10% of tuberculous effusions can be neutrophil predominant [42]. Eosinophilic effusions are defined by PF eosinophils of >10% and are a rarer presentation of pleural effusion. In a large (*n* = 1868), retrospective series by Krenke et al., eosinophilic effusions were seen in 7.2% of all patients with pleural effusion from 1995 to 2007. Malignancy accounted for 34.8% of cases, pleural infection in 19.3%, chest trauma in 8.9%, post-medical or surgical procedure in 4.4%, pneumothorax in 3.7%, and autoimmune in 1.5% [43]. Whilst drugs are often implicated in eosinophilic pleural effusions, it is difficult to be certain of the exact incidence and prevalence as most of the evidence has come exclusively from case reports [44]. Even across a meta-analysis of eosinophilic pleural effusions by Oba and Abu-Salah with 687 cases, there was not a single case of drug-induced eosinophilic effusion recorded. The authors concluded that many studies may have incorrectly classified this important group as “idiopathic eosinophilic effusions” [45]. Diagnosis is fraught with difficulty and relies upon observing the behaviour of the effusion following cessation of offending agents. Whilst the list of offending agents is not exhaustive, it does continue to grow and clinicians must be familiar with them [46].

Diagnosing an MPE through PF poses some challenges. Historically, PF cytology yields were suggested to be 60%; however, more recent data would suggest that this is likely an overestimation [13,47,48,49]. It is suggested that 50–75 mL should be sent as a minimum to maximise yield and a repeat fluid cytology may increase the yield by a further 26%, though this latter claim is based on a small retrospective series [50,51]. There exists a heterogeneity for yield, varying according to underlying tumour type. Typically, ovarian and breast cancer have a high diagnostic yield in PF (94.7% and 70.7%, respectively), lung adenocarcinoma fares the best of the lung cancers (82%), whilst mesothelioma fares particularly poorly (6.1%) [47].

Diagnosing malignant pleural mesothelioma (MPM) can prove difficult. Many MPMs do not exfoliate tumour cells into the PF and this is especially true for the sarcomatoid subtype. The presence of reactive epithelioid mesothelial cells should not necessarily reassure clinicians of a benign aetiology. Whilst there are some cytological features that may raise the suspicion of MPM—extent of mesothelial proliferation, presence of papillary structures, scalloped borders of cell clumps, intercellular windows, variation of cytoplasmic staining and its density, and low nuclear-to-cytoplasmic ratios—these features may also be found in reactive epithelioid mesothelial cells [52]. When adequate cellular material has been obtained, certain immunocytochemistry patterns can be useful in diagnosing MPM over benign disease. The homozygous deletion of BRCA-1 protein (loss of BAP1) shows 100% specificity for differentiating malignant mesothelial proliferation from benign. In the presence of BAP1, deletions of p16 are seen in up to 80% of MPM (especially sarcomatoid subtypes) [53,54]. In spite of these advances, demonstrating tissue invasion histologically (into the chest wall soft tissue or underlying lung parenchyma) remains the most reliable indicator of malignant pleural disease and therefore, the authors would always advocate securing a histological diagnosis over relying on a cytological one where possible. Once malignant pleural disease has been confirmed, a further difficulty encountered is differentiating MPM from secondary tumours involving the pleural. The current BTS guidance on the investigation of MPM suggests a combination of at least two positive mesothelial immunohistochemistry markers (e.g., calretinin, cytokeratin 5/6, Wilms tumour 1, D-240) with at least two negative lung adenocarcinoma immunohistochemistry markers (e.g., TTF1, CEA, Ber-EP4), which is the cancer subtype MPM is often confused with [55].

Importantly, in this modern age of targeted and personalised therapy, it is important to consider the definition of “yield” being more than diagnosis alone, but also whether this is sufficient to direct management. Many patients who were previously not eligible for systemic anti-cancer treatments are now being offered disease modifying therapies and therefore, the case for diagnostic confirmation of malignant pleural disease continues to grow. Many novel oncological therapies are predicated on identifying molecular markers and subsequently, adequacy of samples is also a growing issue. Simply demonstrating the presence of malignant cells is no longer sufficient; adequate cellular material is required to perform the necessary molecular diagnostics. It has been demonstrated that a positive PF cytology result is not enough to affect a change in management and strong cases have been made for alternative diagnostic pathways for these patients (see thoracoscopic biopsies) [48,49].

## 6. Pleural Biopsies

In view of the shortcomings of PF cytology, pleural biopsies remain the gold standard for diagnosis of malignant pleural disease. They have an established role in the diagnosis of pleural TB and recently, have also found a place in the diagnosis of non-tuberculous pleural infection. Clinicians have a number of options to choose from in their choice of technique for obtaining pleural biopsies and we outline a few of these below.

## 7. Closed Reverse-Bevel Needles (Abrams or Cope)

**Figure 2:**
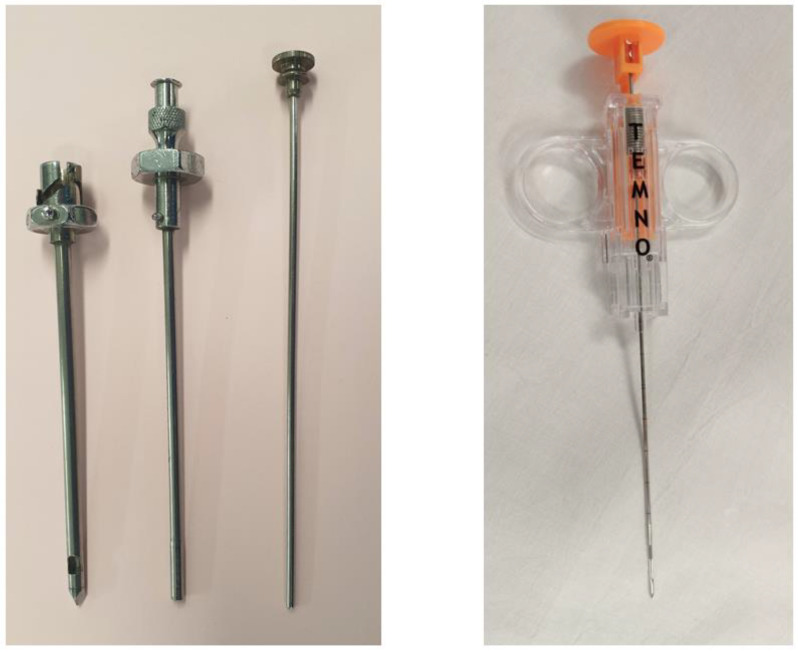
Abrams needles (**left**) and Temno needle (**right**).

Blind biopsies using these eponymous needles (Figure 2) date back to the 1950s when they were first devised. Whilst they have fallen out of practice in many institutions, including our own, they still enjoy utility in many parts of the world. Their diagnostic accuracy varies according to condition. In conditions known to cause diffuse pleural disease, typically tuberculosis, they carry a high yield amongst skilled operators. This has been quoted as high as 90% in the literature [56,57]. Where the technique falls short however (sens < 60%), is with the diagnosis of malignant pleural disease, which has a patchier distribution and tends to favour regions not easily accessible percutaneously (posteromedial and diaphragmatic regions).

The addition of CT guidance to the Abrams needle technique has been shown to improve sensitivity to 81.8%, increasing to 93% when pleural thickness exceeded 1cm, across all cases of cytology negative exudates in a randomised controlled trial (RCT) by Metintas et al. [58]. These needles are larger than their cutting needle counterparts (Figure 2) and as such, come with significant rates of complications: pain 15%, iatrogenic pneumothorax 15%, bleeding < 2% [13].

## 8. Core Cutting Needle Biopsy

In the above cited RCT, the core cutting needle biopsy was suggested to be inferior (sens 67%) compared to an Abrams needle; however, a direct comparison is difficult. The cutting needle biopsy (Tru-Cut) was used with ultrasound prior to insertion (US assisted) rather than real time visualisation of the needle (US-guided), whilst the Abrams needle was inserted under CT guidance. Where direct head-to-head comparisons have been performed, these suggested that US-assisted techniques favoured the Abrams needle over the Tru-Cut needles in diagnosing pleural TB (sens 81.8% vs. 65.2%). Results from this non-inferiority study are underpowered and open to bias however, as recruitment was terminated early, citing patient safety [59]. Just 89 patients were recruited from a planned 220, following a pre-planned interim analysis that suggested the yield from the Tru-Cut was lower than the original estimates investigators had based power calculations on [60].

Studies that have looked at the diagnostic accuracy of core cutting needles using a US-guided procedure suggest diagnostic accuracy rates closer to 90%, though they are retrospective in nature [61,62]. This would all suggest that diagnostic accuracy hinges more on the use of image guidance rather than the choice of needle. Therefore, by extension, it would not be unreasonable to suggest larger reverse-bevel needles would have a greater yield in blind biopsies for diffuse pleural disease compared to their cutting needle counterparts, but once image guidance is employed, this effect is attenuated [63].

A role for image-guided cutting needle biopsies in pleural infection has recently been brought to light through the recent AUDIO study. This was a feasibility pilot study where patients with a confirmed diagnosis of pleural infection following a diagnostic aspirate went on to have a US-guided Temno (Figure 2) cutting needle biopsy in the same sitting as an intercostal drain. The pleural biopsies produced a higher diagnostic yield than either PF or blood cultures (45% vs. 20% vs. 10%), with an exaggerated difference in patients already receiving antibiotics (40% vs. 13% vs. 7%) [64]. A multicentre trial is now planned in order to test this hypothesis.

## 9. Ultrasound vs. CT-Guided

The literature comparing ultrasound-guided to CT-guided biopsies is sparse. A recent meta-analysis across seven studies and 165 patients with US-guided pleural biopsies across both cutting needles and reverse-bevel needles suggested a pooled sensitivity of 83% (95% CI 75–89%) with rates of pneumothorax at 3.6%, wound infection 3%, and empyema < 1% [65]. This is similar to the sensitivity suggested by Metintas et al. for their CT-guided Abrams needle technique.

**Figure 3:**
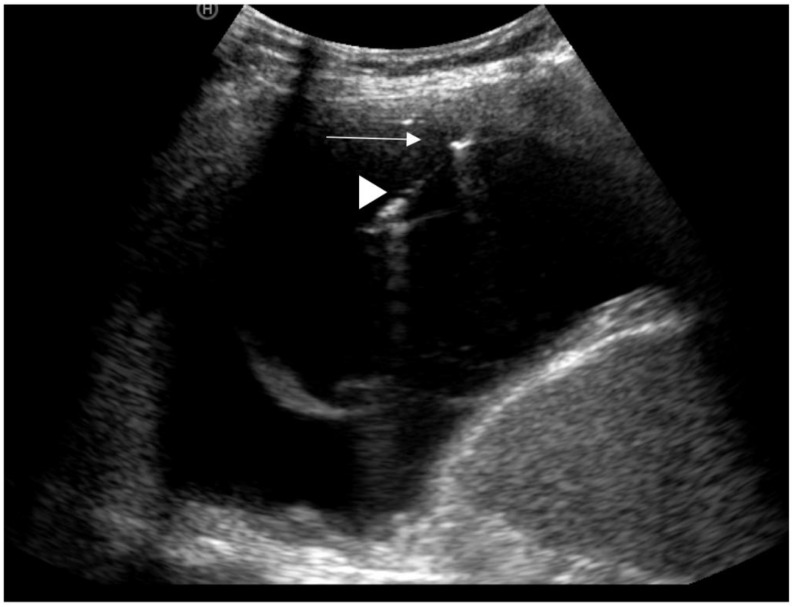
Image of a US-guided needle biopsy; white arrowhead = needle, white arrow = pleural thickening.

**Figure 4:**
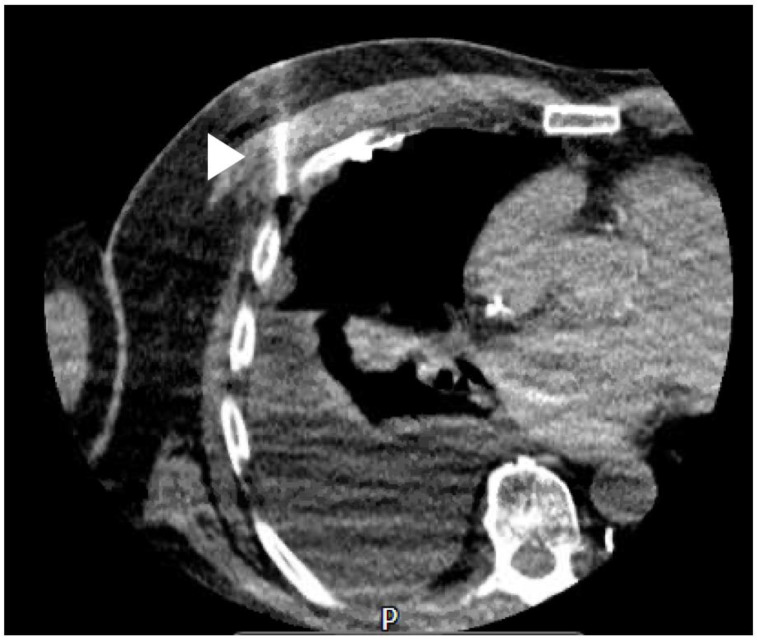
Image of a CT-guided needle biopsy; white arrow = needle.

A large retrospective series across 273 patients suggested there was little difference in diagnostic accuracy between the two techniques (technical success of 97.1% in the US-guided group vs. 96.5% in the CT-guided group). The series included both pleural-based lesions as well as peripheral lung lesions, highlighting additional utility of the US approach. Importantly, this study also concluded that US-guided procedures were quicker, cheaper, and had a lower risk of iatrogenic pneumothorax (14.7% vs. 5.8%) [66]. In practice, the availability of skilled operators in either technique is likely to be the rate limiting step in choice of test. Traditionally, CT-guided procedures have been within the remit of radiologists, whilst US-guided procedures are increasingly being delivered by respiratory physicians. Both techniques have their advantages and disadvantages. Thoracic US uses non-ionising radiation, is quicker to use, and allows for the operator to react to respiratory motion in real-time without reliance on breath-holding techniques, which some patients may be unable to perform. In contrast, CT can be used to target lesions that would not be identifiable on US (shielded beneath bony structures or at depth) (Figure 3 and Figure 4).

At this point, it is worth mentioning the role of PET-CT. The TARGET study set out to specifically assess the role of PET-CT-guided biopsies in patients with ongoing suspicion of pleural malignancy despite a negative CT-guided biopsy. This was built on the premise that malignant pleural mesothelioma (MPM) in particular proves diagnostically challenging given its radiological appearance and the degree of overlap with benign pleural thickening [67]. Surprising to most pleural physicians and radiologists, their primary outcome of pleural malignancy correctly identified on 2^nd^ biopsy was not met (presented at BTS Winter 2019); however, the trial is still pending full publication and there is certainly more to learn from this.

## 10. Thoracoscopic Biopsies

Pleural biopsies performed via an endoscopic approach (thoracoscopy) under direct visualisation are considered the gold standard for diagnosing an unexplained pleural effusion and particularly useful in diagnosing malignant pleural disease. Thoracoscopic biopsies can be performed through the Local Anaesthetic Thoracoscopy (LAT) approach, also entitled Medical Thoracoscopy (MT), often through a single port or the more invasive Video-Assisted Thoracoscopic Surgical (VATS) approach, using up to three ports. LATs are usually performed by physicians in an awake patient, spontaneously breathing under sedation whereas VATS are usually performed by thoracic surgeons in an anaesthetised patient with single-lung ventilation. Whilst both techniques allow for visual inspection of the pleural cavity, performing both a diagnostic and therapeutic procedure in the same sitting (to achieve long-term effusion control through pleurodesis), LAT does have some limitations. In order to safely insert the thoracoscopic port and other instruments, adequate access within the pleural space is required. In the presence of moderate-large pleural effusions, this is straightforward; however, where this is not the case, it may be necessary to induce an artificial pneumothorax through the use of a Boutin needle. This technique is both safe and highly effective in the hands of skilled operators in enabling LAT in patients with inadequate PF. In a series of 77 consecutive patients in whom this was attempted, in 67 (87%), the operators were able to proceed with LAT with no adverse events reported [68]. The ability for the lung to collapse down on Boutin needle induction is heavily dependent on the presence or absence of adhesions between the visceral and parietal pleura, a feature common to malignant pleural disease. Pre-procedural thoracic US has been shown to be quite effective at detecting a lung that is unlikely to collapse on Boutin needle induction, by way of detecting “lung sliding” [63,69]. In these situations, opting for a VATS approach that the surgeon may convert to an open thoracotomy or an image-guided approach are alternative options, depending on patient suitability.

Both approaches are suggested to have a diagnostic sensitivity exceeding 90% in detecting malignant pleural disease. The 2010 BTS guidelines pooled results across 22 case series to demonstrate a diagnostic sensitivity of 92.6% in diagnosing malignant pleural disease via LAT [70]. A similar diagnostic sensitivity rate was also observed in an RCT comparing LAT to CT-guided Abrams needle biopsy (94.1% vs. 87.5%) [71]. For VATS approaches, diagnostic sensitivities of 89–95% have been quoted in the literature for diagnosing malignant pleural disease [72,73].

LAT is generally considered a safe procedure; across 47 studies and 4756 patients, major complications were reported in 1.8% of cases, minor complications in 7.8%, and mortality in 0.34% [70]. Major complications consisted of empyema, haemorrhage, port-site tumour growth, bronchopleural fistula, persistent air leak, and pneumonia. Minor complications consisted of subcutaneous emphysema, minor haemorrhage, operative skin site infection, hypotension peri-procedure, fever, and atrial fibrillation. Across these studies, no deaths were observed in diagnostic thoracoscopies alone (0/2421) and were all seen in the therapeutic thoracoscopy arm (16/2315). Nine out of sixteen were seen in a single randomised control trial, attributed to the use of non-graded talc leading to unintended absorption and toxicity, with resultant acute respiratory distress syndrome and respiratory failure [74]. As a result, best practice is now for the use of graded talc to avoid such complications and this approach has been validated in a large prospective multicentre cohort study [75].

VATS is considered more invasive and patients by definition need to be fit enough to survive a general anaesthetic and therefore, in comparing complication rates between LAT and VATS, it must be understood that the patient groups are different. Reported complication rates in VATS vary; in one series across 185 patients, 15% were reported to have had a major complication whilst in another across 86 patients, the major complication rate was just 1.2% [73,76]. A recent retrospective review of patients undergoing LAT (described as “awake thoracoscopy” through a single port) and VATS were compared in a single centre and the rates of major complications were similar (LAT 2.6% vs. VATS 4%) but cost was significantly lower in the LAT group. However, as suggested above, there were significant baseline differences in the patient characteristics between groups [77]. True head-to-head comparator trials that hold clinical meaning for LAT against a VATS approach in diagnosing malignant pleural disease are unlikely to occur (due to patient selection bias and therefore, applicability).

There remains some debate over the use of rigid (RT) vs. semi-rigid thoracoscopes (SRT) and more recently, the rigid mini-thoracoscope (RMT) has joined the fray. RTs allow for larger biopsies, given their larger working channels, and this may facilitate deeper pleural biopsies, which contain fat and skeletal muscle. This allows it to overcome some of the difficulties presented by a densely thickened or fibrotic pleura, which can result in false negative biopsies [78]. This becomes more relevant when the leading diagnosis is MPM and the degree of invasion provides both diagnostic and prognostic information. Head-to-head trials comparing all three are lacking. Similar diagnostic yields between RT vs. SRT have been reported in a retrospective case series (96.3% vs. 92.3%) [79]. These yields are reproduced in both a systematic review and meta-analysis of SRT [80,81]. In the MINT study, a single centre RCT comparing RMT to SRT, the authors did find a greater diagnostic yield in the SRT group (81.1% vs. 69.4%) [82]. However, the results lacked statistical significance and this is likely due to the small sample size. Operator expertise with this novel technology compared to the more familiar SRT may have also affected their measured outcomes [78].

The take-home message from all of these studies is perhaps, the thoracoscopic approach has extremely high diagnostic yield, irrespective of device or operator, physician or surgeon. It is preferable to start with an awake thoracoscopic procedure where possible and reserve a procedure under general anaesthesia with single lung ventilation for those in whom the alternative is not technically feasible.

## 11. Imaging

Whilst the use of imaging in specifically targeting biopsies has been explored, there are some wider points around the role of imaging as a diagnostic tool to discuss.

## 12. Chest Radiograph

**Figure 5:**
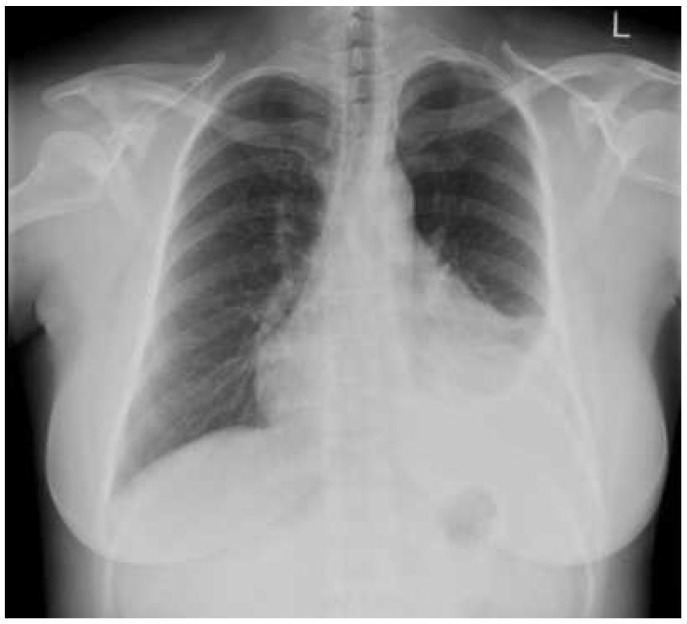
Chest radiograph (CXR) demonstrating a pleural effusion.

This modality has largely withstood the test of time and still remains the most easily accessible form of chest and pleural imaging, worldwide. It will often be the initial imaging performed for any patient with suspected pleural disease. Whilst higher quality images are obtained by performing the radiograph in a posteroanterior (PA) projection, often in emergencies, this is not possible. Chest radiographs (CXR) performed in the supine anteroposterior position are less sensitive in detecting pleural air or fluid. It has been estimated that a pleural effusion of approximately 200 mL in volume would be visible on an PA CXR (Figure 5), whilst a smaller volume of 50 ml would be detectable on a lateral CXR [83].

## 13. Ultrasound

**Figure 6:**
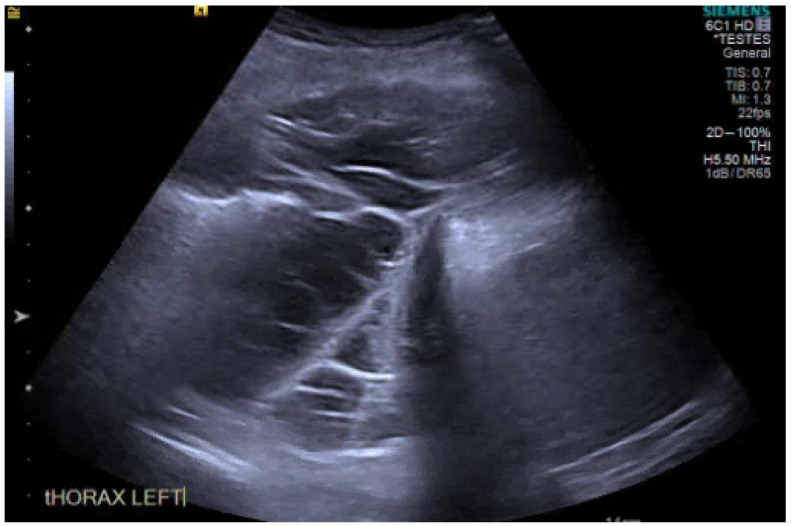
Thoracic ultrasound (TUS) image of a septated effusion.

Thoracic ultrasound (TUS) has revolutionised the diagnosis and delivery of care in pleural disease. TUS is far more sensitive at detecting pleural effusions than CXR, being able to detect even just 3–5 ml of pleural effusion and >100 mL of effusion with a sensitivity of 100% [84,85]. Furthermore, TUS allows for better characterisation of an effusion, for example the degree of echogenicity or septations (Figure 6). In fact, TUS has much greater sensitivity for identifying septations within an effusion compared to computed tomography (CT) [86]. However, interpretation of these findings is not always clear cut. Whilst convention would suggest a hyperechoic, septated effusion must be an exudate and indeed, there is some evidence to support this, these rules are not absolute. Across 320 patients with both transudates and exudates, this prospective observational series by Yang, now 28 years old, suggested transudates were always anechoic in appearance whereas exudates had a variety of appearances across a spectrum of echogenicity, including anechoic [87]. This assertion that all transudates must be anechoic has been refuted with recent evidence to the contrary. Asciak and colleagues demonstrated in their own prospective series that the specificity of “echogenicity” in identifying an exudative effusion over a transudate was only 57.1% [88]. In their series across 140 cases, they identified six (7%) patients with echogenic effusions that were ultimately diagnosed as a transudate. Their finding is supported by other retrospective work and questions some assumptions we have made about TUS [89,90].

The role of TUS goes beyond recognition and classification of effusions. In a study mirroring some of the CT characteristics of malignant pleural disease by Leung et al., Qureshi and colleagues demonstrated the presence of diaphragmatic and parietal pleural nodularity, parietal pleural thickening > 1 cm, and hepatic metastases in diagnosing malignant pleural disease has a sensitivity of 73% and specificity of 100% [91,92].

There has been increasing enthusiasm recently in the role of TUS in diagnosing pneumothorax, perhaps more so by the emergency and critical care world. Detecting the absence of “lung sliding” on B-mode and loss of the “sea-shore” sign on M-mode is a more sensitive tool than a supine CXR. However, care must be taken in differentiating a pneumothorax from bullous emphysema or prior pleurodesis. As of yet, there is no role for TUS in quantifying the size of a pneumothorax or for procedural guidance in this condition [90]. The authors’ view is that the use of TUS in detecting pneumothorax lies solely in the urgent/trauma setting and where there is any doubt on CXR, a CT scan is the next investigation of choice.

The ability of TUS to identify lung sliding has also highlighted a role for its use in predicting pleurodesis success. Corcoran and colleagues used a “pleural adhesion” score (based on the presence or absence of lung sliding) to estimate pleurodesis success after talc slurry instillation. They found that a lower score correlated with failure [93]. A multicentre randomised controlled trial is now “in submission” (SIMPLE, ISRCTN 16441661) to validate these findings and to determine if a TUS-directed approach to pleurodesis in MPE results in a shortened length of stay when compared to daily CXR [94].

Finally, TUS can be used to predict non-expansile lung prior to pleural intervention. Salomonsen and colleagues demonstrated that in cases of entrapped lung, both the motion and strain related to the transmission of the cardiac impulse through an atelectatic segment of lower lobe measured during both M-mode and speckle-tracking imaging fared better at predicting entrapped lung compared to pleural elastance measurement [95]. At present, this technique is likely to be available only to advanced US operators and is still awaiting multisite validation.

## 14. CT

**Figure 7:**
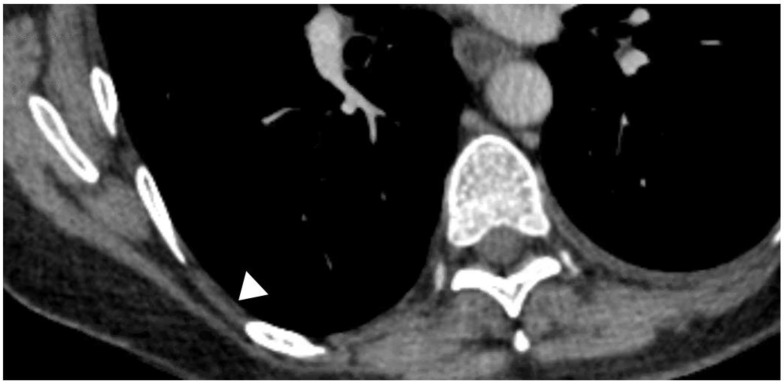
CT appearances of the “pleural stripe”; (white arrow head)

Whilst TUS has its many uses, there is no replacement for cross-sectional imaging which can provide a three-dimensional reconstruction of the chest and pleural cavity in a way TUS might struggle to, except in the hand of the most skilled operators. In health, it is difficult to visualise the pleura on CT scan and the “intercostal stripe” is often a surrogate; it consists of visceral and parietal pleura, extrapleural fat, endothoracic fascia, and the innermost intercostal muscles (Figure 7) [96].

To optimise CT imaging of the pleura, iodinated intravenous contrast is recommended and ideally, a venous phase or “Pleural phase” scan 60–90 s post infusion should be taken. Failure to achieve a “Pleural phase” scan has been shown to result in poorer diagnostic yields [97,98]. PF drainage prior to imaging is not a prerequisite and in a series across 32 patients with pre and post drainage CTs, the second scan did not provide any new information to influence clinical management [99].

Whilst it can be argued that all patients with complex pleural pathology, whether that be tethered pneumothoraces, unexplained effusions, diffuse pleural thickening, broncho-pleural fistulae, or any other relevant thoracic pathology (e.g., lung abscess, oesophageal leak, etc.) should have cross-sectional imaging, where CT really proves essential is in the diagnosis and management of late-stage empyema and malignant pleural disease.

**Figure 8:**
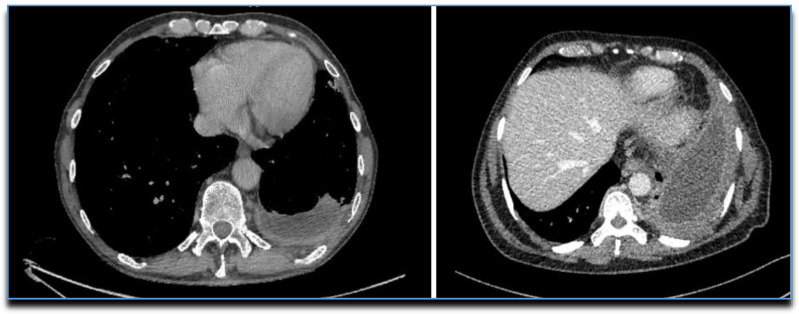
CTs showing the “Split Pleura” sign.

Whilst TUS can identify septations within an effusion better than CT, in cases of advanced empyema with a non-draining collection, CT can be used to check drain position and plan for thoracic surgical intervention. The “split pleura” sign (Figure 8) and the presence of >30 mm distance between the parietal and visceral pleura were shown to correctly identify a complex parapneumonic pleural effusion (CPPE) from a simple PPE with a sensitivity of 79.4% and specificity of 80.9% [100,101]. A number of other features are also seen in empyema, though they are not specific to the condition and can represent a PPE too: contrast enhancement of the pleura, thickened parietal pleura, increased attenuation and/or thickness of extra pleural subcostal fat, gas bubbles suspended within PF, or loculation of PF [96].

**Figure 9:**
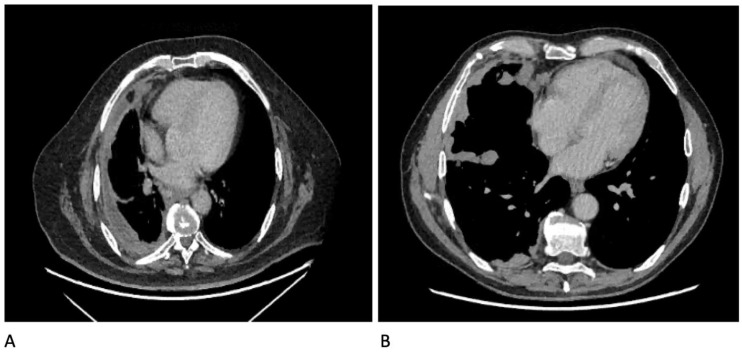
CT images showing some features of malignant pleural disease: (**A**) Circumferential pleural thickening; (**B**) Nodular pleural thickening involving mediastinal surfaces and fissures.

The CT features of malignant pleural disease have been derived through a number of small retrospective studies; though not the largest, the series by Leung et al. was the earliest and remains the most recognised and cited. These studies largely agree that the following features on CT are more suggestive of malignant disease than benign (Figure 9): nodular pleural thickening (sens 38–53%, spec 87–100%), pleural thickening along mediastinal surfaces (sens 14–74%, spec 83–97%), thickening of the parietal pleura >1 cm (sens 36–57%, spec 64–94%), and circumferential pleural thickening encasing the lung (sens 8–54%, spec 63–100%) [92,96,98,102,103,104,105]. However, CT is not the be-all and end-all when it comes to securing a diagnosis of pleural malignancy. Both Tsim et al. and Hallifax et al. demonstrated that the negative predictive value of CT in detecting malignant pleural disease sits somewhere between 54 and 65% and that therefore, there are a significant number of patients with malignant disease who have a “benign” CT (1 in every 2–3 cases) [97,106]. Therefore, where the pre-test probability is high enough, more definitive investigations should be undertaken (the current gold standard for which is thoracoscopy). The utility of CT might be increased further by also imaging the abdomen and pelvis. Whilst this is standard of care for all patients who enter a cancer pathway, it is not necessarily the first choice of investigation for an unexplained effusion. Syer et al. has recently published the results from an observational series of 249 patients presenting with a unilateral effusion, in whom clinically significant findings were identified beneath the diaphragm in 59 patients (24%). They define clinically significant as a finding that either identified the primary diagnosis (identified the primary tumour in 6.8%), upstaged any malignant disease (12.9%), or highlighted a favourable site for further investigation (alternative biopsy site in 2%) [107].

## 15. Other Diagnostic Tests

Pleural manometry (PM) describes the measurement of intrapleural pressures using a water or digital manometer. Studies measuring the change in intrapleural pressure during thoracocentesis, to derive a measure of pleural elastance and therefore, predict non-expansile lung, have produced mixed results. Chopra et al. demonstrated that although patients with elevated pleural elastance were less likely to achieve lung expansion (OR 6.3 of achieving lung re-expansion on CXR if normal pleural elastance), there was a degree of discordance in this relationship as some 28% of patients with lung re-expansion also had elevated pleural elastance [108].

The Pre-EDIT trial was a feasibility study randomising patients into pleural elastance-driven therapy (indwelling pleural catheter vs. talc slurry pleurodesis via chest drain) against standard care of chest drain with a view to talc slurry pleurodesis if lung expansion was achieved. As a feasibility study, the authors demonstrated the suitability for a phase 3 study [109]. Though not powered to demonstrate any difference, across small patient numbers, a high pleural elastance (seven patients) showed a sensitivity of 100% and specificity of 67% for non-expansile lung.

Lentz and colleagues explored the use of PM as an aid to preventing pleural pressure-related complications in large-volume thoracocentesis against a symptom-guided approach in a multisite randomised controlled trial. They found no difference in their primary outcome (chest discomfort) [110].

PM also has utility in pneumothorax assessment. The mechanisms underpinning PM measurements in pleural effusion and pneumothorax do vary and lessons learnt from one cannot be applied in the other. Heidecker et al. were able to use PM to differentiate between pneumothorax ex vacuo (“stable”) from iatrogenic pneumothorax (“unstable”) during a pleural procedure. “Stable” or “pressure-dependent” pneumothoraces (seen in non-expansile lung) bear the hallmark of a stable pleural pressure when the chest drain is clamped as opposed to the rising pleural pressure during clamping that is seen in an “unstable pneumothorax” (traumatic or spontaneous pneumothoraces) [111].

Whilst there is physiological plausibility in using pleural manometry as a diagnostic tool and to guide treatments, as of yet there is no compelling evidence for its routine use in the management of pleural disease. With the advent of further studies, this may change [112].

## 16. Conclusions

Whilst the topic of “Diagnostics” in pleural disease is an expansive field and one that is ever growing, basic principles hold true and no single test will ever provide the complete answer. It is through a thorough history, clinical assessment, evaluation of pre-test probabilities, and careful selection of diagnostic tests, of which there are many, can the physician be confident in their diagnosis. Whilst conventional wisdom would suggest starting with the least invasive diagnostic tools, mounting evidence points to accelerated diagnostic pathways with greater clinical efficacy as the future direction of travel in pleural disease.

## Figures and Tables

**Table 1:** Causes of pleural effusions [10].

Transudative Effusions	Exudative Effusions
Congestive cardiac failure	Parapneumonic
Cirrhosis	TB pleuritis
Nephrotic syndrome	Primary or secondary thoracic malignancy
Glomerulonephritis	Pulmonary embolism
Peritoneal dialysis	Pancreatitis
Hypoalbuminaemia	Post myocardial infarction
Cerebrospinal fluid leak	Collagen vascular disorders
Urinothorax	Drug-related
	Haemothorax
	Chylothorax
	Benign asbestos-related pleural effusions

**Table 2:** Minimum standard assays for PF.

Assay
Biochemistry panel: Protein, LDH, Glucose, pH
Microbiology panel: Gram stain + Culture
Pathology panel: Cytology for differential cell count + abnormal cells

**Table 3:** Typical PF biochemical patterns [8,13,34,35].

Condition	Typical PF Biochemical Patterns
Cardiac failure	Low Protein, Low LDH, N terminal pro-brain natriuretic peptide (NT-proBNP), but closely mirrors serum NT-proBNP
Pleural infection	High Protein, High LDH (>1000), very low Glucose, Low pH
Malignant pleural effusion	High Protein, High LDH, (Low Glucose)
Rheumatoid effusion	Very low Glucose
TB effusion	Very high Protein, low glucose
Dural leak	Very low Protein
Urinothorax	Very low Protein, PF/Serum creatinine ratio > 1, pH < 7.30
Pancreatitis	PF/serum amylase ratio >1, PF amylase > upper limit of normal serum levels
Chylothorax	Elevated Triglycerides (>1.24 mmol/L), Chylomicrons
Pseudochylothorax	Elevated cholesterol (>5.18 mmol/L), cholesterol crystals
Haemothorax	PF haematocrit/Serum haematocrit > 0.5
